# Supplementary material for: Epigenetic regulation of serine biosynthesis by PHF8 during neurogenesis
Source: EMBO Rep. 2026 Feb 19;27(6):1540–60. doi: 10.1038/s44319-026-00713-8 (PMC13022353; doi:10.1038/s44319-026-00713-8)
Supplement: Supplementary file 1 — Appendix [file 44319_2026_713_MOESM1_ESM.pdf]

Appendix for

Epigenetic Regulation of Serine Biosynthesis by PHF8 During Neurogenesis

Table of contents:

Appendix Figure S1________________________Page 2

Appendix Figure S2________________________Page 3

Appendix Figure S3________________________Page 4

Appendix Figure S4________________________Page 5

Appendix Figure S5________________________Page 6

Appendix Figure S6________________________Page 8

Appendix Figure S7________________________Page 8

Appendix Table S1 ________________________Page 9

Appendix Table S2 ________________________Page 10

APPENDIX FIGURES

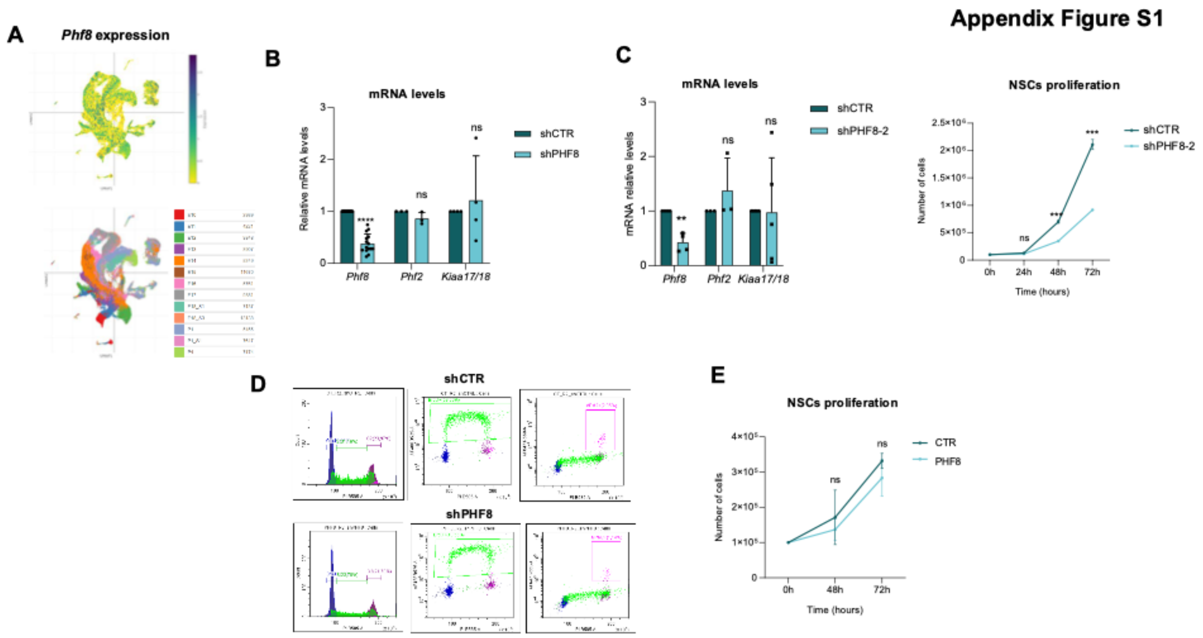

Appendix Figure S1. PHF8 maintains neural stem cells proliferation

(A) UMAP plot showing PHF8 expression in the developing mouse cerebral cortex. Data are from publicly available single-cell RNA-seq experiments (link to dataset).

(B) NSCs were infected with lentivirus expressing shRNA control (shCTR) or shRNA specific for PHF8 (shPHF8). 48 h post-infection, total RNA was extracted and transcript levels of *Phf8*, *Phf2* and *Kiaa17/18* were measured by qPCR. Data represent the mean of at least three biological independent experiments, each performed in triplicate. Errors bars represent SD. ****p<0.0001; ns, not significant (Student's t-test).

(C) NSCs were infected with lentivirus expressing a shRNA control (shCTR) or a second PHF8-specific shRNA (shPHF8-2). Total RNA was purified to assess PHF8 mRNA levels via qPCR. mRNA expression values were normalized to the housekeeping gene *Gapdh*. Data represent the mean of at least three biological independent experiments. Errors bars represent SD. **p<0.01; ns, not significant (Student's t-test) (as shown in the left part of the figure). Growth curve showing proliferation of NSCs infected with shCTR or shPHF8-2 lentivirus over 72 h. Data represent the mean of three biological independent experiments

performed in triplicated. Errors bars represent SD. ***p<0.001 (Student's t-test) (right part of the figure).

(D) Flow-cytometry data of shCTR and shPHF8 NSCs previously stained with 5'-bromo-2'-deoxyutidine (BrdU) and MPM2.

(E) NSCs were infected with a lentivirus expressing PHF8 under doxycycline control. Doxycycline (Dox) was added (PHF8) or omitted (CTR), and cell proliferation was assessed 72 h later using growth curves. Data represent the mean ± SD of three independent biological experiments, each performed in triplicate. Error bars indicate SD. ns, not significant (Student's t-test).

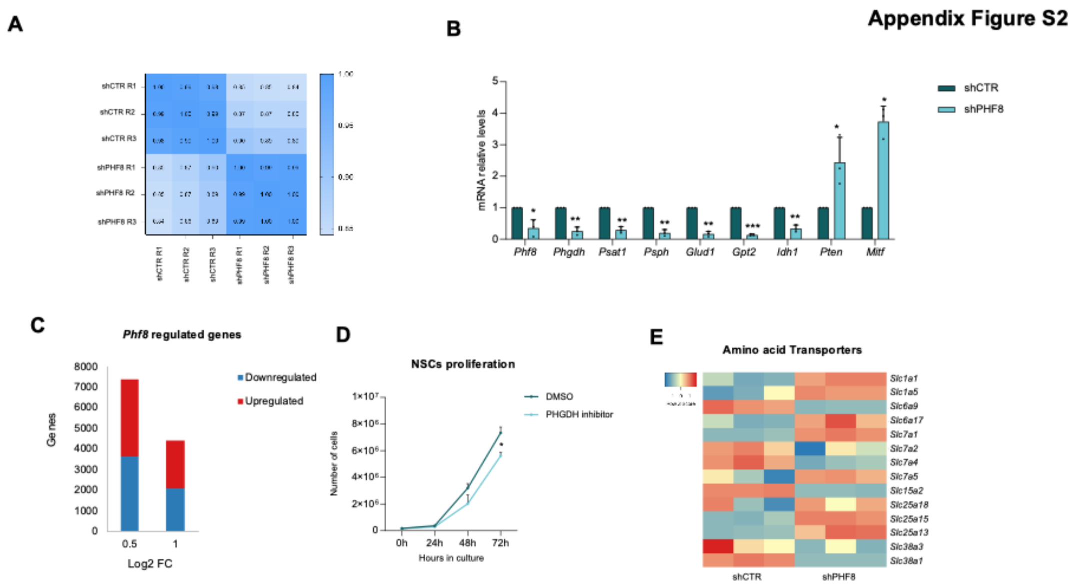

Appendix Figure S2. PHF8 regulates transcription of Serine Biosynthesis Pathway (SBP) genes

(A) Clustered heat-map showing Pearson correlation of RNA-seq samples from shCTR and shPHF8 NSCs, based on transcriptome-wide coverage.

(B) RNA-seq results from shCTR and shPHF8 NSCs were validated by qPCR for the indicated genes. Expression values were normalized to the housekeeping gene *Gapdh* and

are presented relative to shCTR NSCs. Data represent the mean of three biological independent experiments. Errors bars represent SD. *$p<0.05$; **$p<0.01$; ***$p<0.001$, (Student’s t-test).

(C) Bar graph showing the number of upregulated and downregulated genes in shPHF8 vs. shCTR NSCs, filtered by p-value < 0.05 and ranked by increasing log_2 fold change.

(D) Growth curve illustrating the proliferation of NSCs treated with DMSO or PHGDH inhibitor over 72 h. Data represent the mean of three independent biological experiments performed in triplicated. Errors bars represent SD. *$p<0.05$ (Student’s t-test).

(E) Heat-map of differentially expressed amino acid transporter genes identified by RNA-seq in shCTR and shPHF8 NSCs. Only genes with p-value<0,05 and absolute log_2 fold change (FC)>0,5 and (FC)<-0,5 are shown. Three biological replicates were analyzed for the shCTR and shPHF8 condition.

Appendix Figure S3

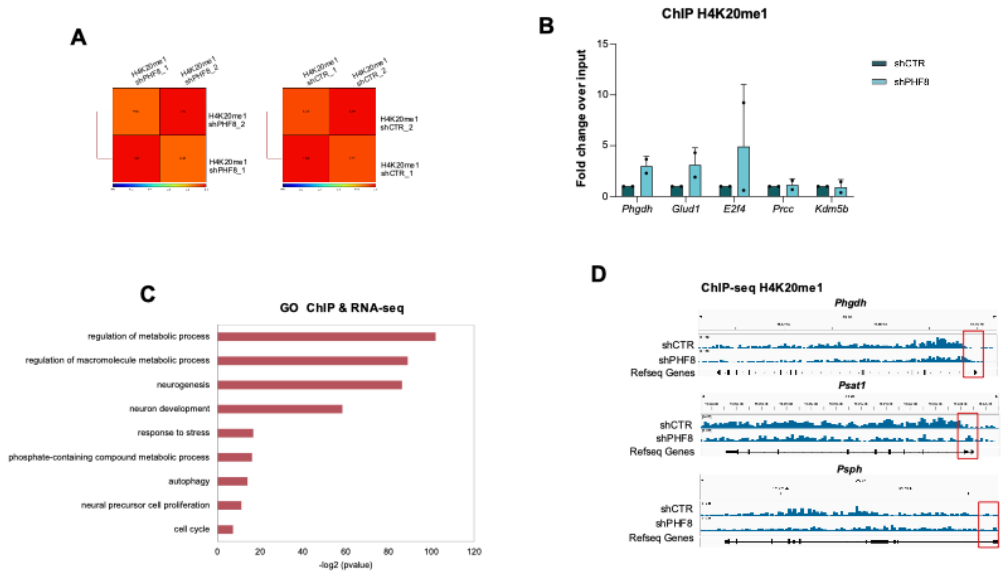

Appendix Figure S3. PHF8 maintains low levels of H4K20me1 at SBP gene promoters

(A) Clustered heatmap depicting Spearman correlation of H4K20me1 ChIP-seq datasets in shCTR and shPHF8 NSCs. The correlation analysis is conducted based on read coverage within consecutive 10-kb genomic regions.

(B) H4K20me1 ChIPs in shCTR and shPHF8 NSCs were analyzed by qPCR at the indicated gene promoters. qPCR data were normalized to the input and presented relative to shCTR NSCs. Results from two biological independent ChIP experiments and technical triplicates are shown. Errors bars represent SD.

(C) Gene ontology (GO) analysis of Biological Process enriched among genes downregulated in the RNA-seq dataset (p-value < 0.05, $\log_2$ fold change > 0.5 or < -0.5) and showing increased H4K20me1 levels upon PHF8 depletion.

(D) IGV snapshots displaying H4K20me1 levels (normalized to input) at *Phgdh*, *Psat1* and *Psph* gene promoters in shCTR and shPHF8 NSCs.

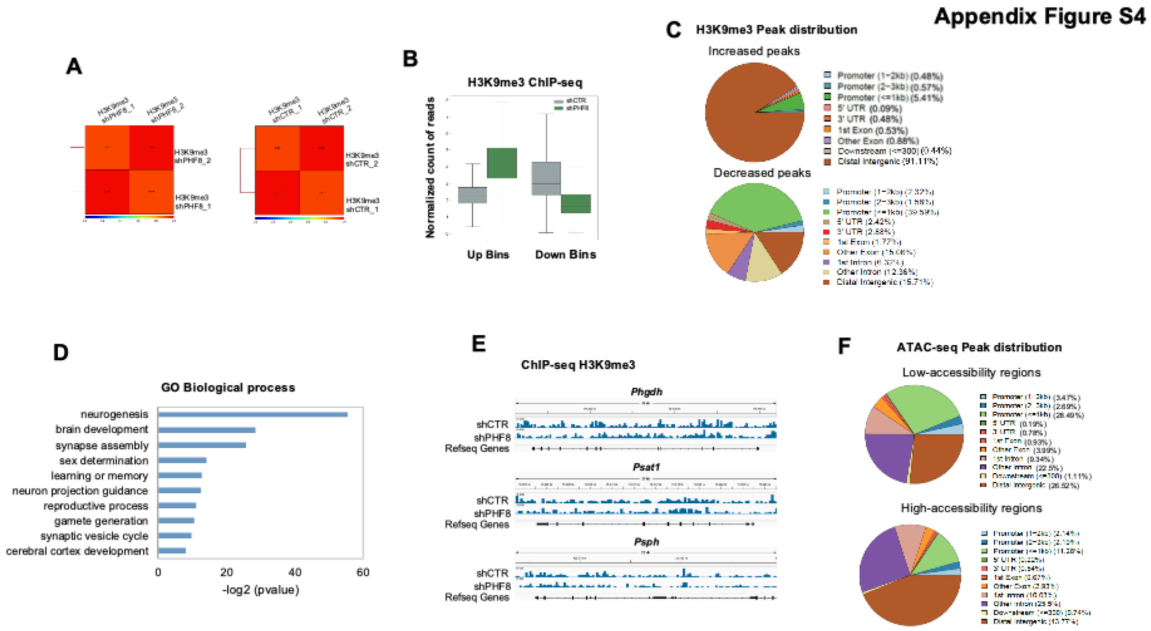

Appendix Figure S4. PHF8 maintains global levels of H3K9me3 (A) Clustered heatmap depicting Spearman correlation of H3K9me3 ChIP-seq datasets in shCTR and shPHF8 NSCs. The correlation analysis is conducted based on read coverage within consecutive 10-kb genomic regions.

(B) H3K9me3 ChIP-seq was performed in duplicate in shPHF8 and shCTR NSCs. The mouse genome was divided into bins of 30 Kb each to assess H3K9me3 enrichment. Box

plots display bins showing gain and loss H3K9me3 in shPHF8 versus shCTR cells. Box plot elements: centerline = median; box limits = 25th and 75th percentiles; whiskers = minimum and maximum.

(C) Genomic annotation of regions with increased H3K9me3 levels upon PHF8 depletion in NSCs.

(D) Gene ontology (GO) analysis of Biological Process enriched among genes regulated by PHF8 (p-value < 0.05, $\log_2$ fold change > 0.5 or < -0.5) and exhibiting increased H3K9me3 levels upon PHF8 depletion.

(E) IGV snapshots displaying H3K9me3 levels (normalized to input) at *Phgdh*, *Psat1* and *Psph* gene promoters in shCTR and shPHF8 NSCs.

(F) Genomic annotation of regions with differential chromatin accessibility (categorized as gains or losses) upon PHF8 depletion in NSCs.

Appendix Figure S5

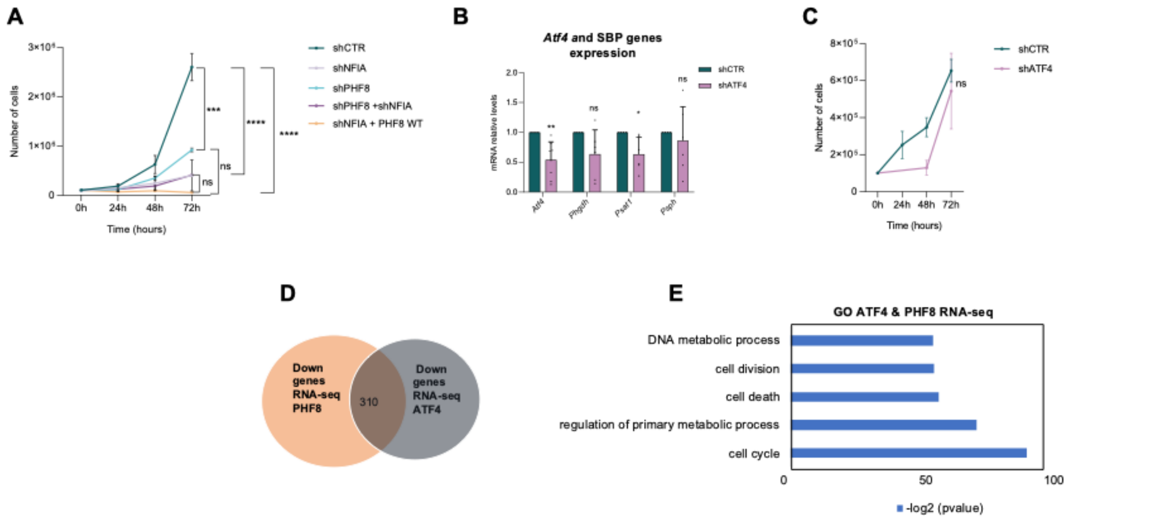

Appendix Figure S5. PHF8 cooperates with Transcription factors to regulate SBP gene transcription

(A) Growth curve showing the proliferation rates of NSCs infected with shCTR or shNFIA lentiviruses over 72 h, in which PHF8 levels were either decreased using shPHF8

or increased through induction with the pInducer vector upon doxycycline addition (see diagram in Fig. 2F). Data represent the mean of three biological independent experiments performed in triplicated. Errors bars represent SD. ***p<0.001; ****p<0.0001 (Student's t-test).

(B) NSCs were infected with lentiviruses expressing shCTR or ATF4-targeting shRNA (shATF4). Total RNA was collected to assess ATF4 mRNA levels of *Atf4*, *Phgdh*, *Psat1*, and *Psph* by qPCR. Expression levels were normalized to *Gapdh*, and data are presented relative to shCTR samples. Data represent the mean of at least four biological independent experiments performed in triplicated. Error bars indicate SD. *p<0.05; **p<0.01; ns, not significant (Student's t-test).

(C) Growth curve showing proliferation rates of NSCs infected with shCTR or shATF4 lentiviruses over 72 h. Data represent the mean of three biological independent experiments performed in triplicated. Errors bars represent SD. ns, not significant (Student's t-test).

(D) Venn diagram showing the overlap between 2032 down-regulated genes identified in the published ATF4 RNA-seq dataset (grey) and the PHF8 RNA-seq dataset from NSCs (Figure 2A) (orange). p-value<0.05

(E) GO analysis highlighting enriched Biological Processes among genes co-regulated by PHF8 and ATF4 identified in (D).

Appendix Figure S6

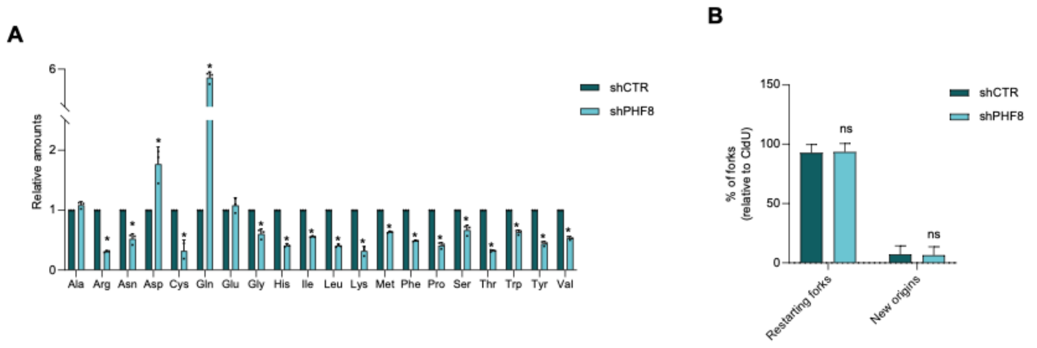

Appendix Figure S6. Metabolic and replication alterations upon PHF8 depletion

(A) Bar diagram showing amino acid levels measured by LC/MS in shCTR and shPHF8 NSCs (n= 3 biological replicates per condition). Statistical significance was determined by one-way ANOVA. Error bars indicate SD. *p<0.05.

(B) DNA fiber assay on shCTR and shPHF8. shCTR and shPHF8 NSCs were exposed for 30 min to CldU (red), followed by 30 min of IdU (green) (n=200). Graphs show % of restart and new replication origins. ns, not significant (Student's t-test).

Appendix Figure S7

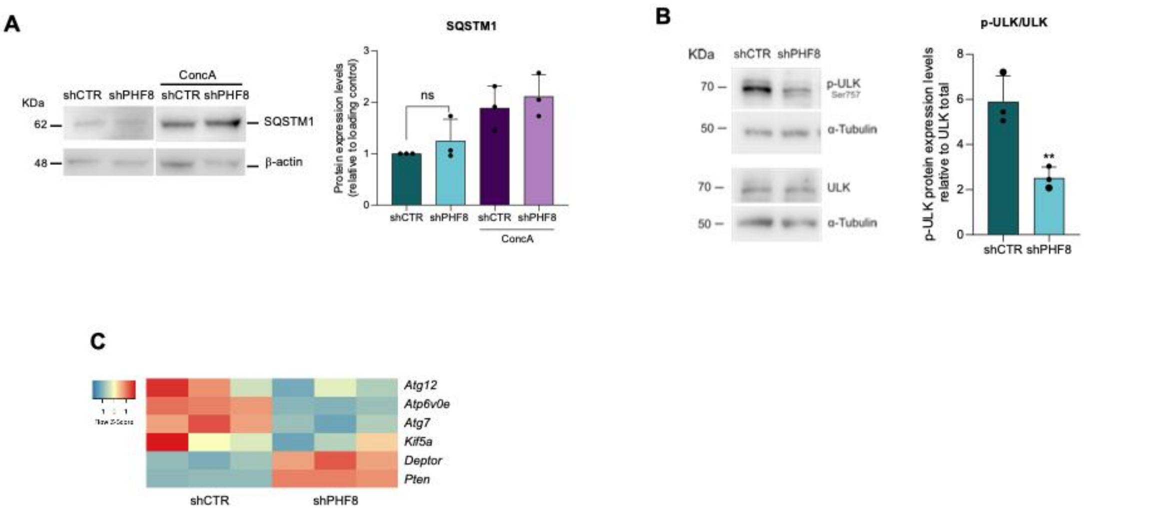

Appendix Figure S7. PHF8 depletion disrupts autophagy

(A) NSCs expressing shCTR or shPHF8 were treated or left untreated with concanamycin A. Total protein extracts were prepared and analyzed by immunoblotting for SQSTM1. The samples derive from the same experiment and that immunoblots were processed in parallel. The same samples were used in Figure 5A. Graphs depict protein expression levels relative to β-actin. Data are representative of three biologically independent experiments. Errors bars represent SD. ns, not significant (Student's t-test).

(B) Total protein extracts were prepared from NSCs expressing shCTR or shPHF8 and analyzed by immunoblotting for ULK and phospho-ULK (p-ULK) (Serine 757). Graphs depict phospho-ULK levels normalized to total ULK. The same samples were used in Figure 5C. The same loading control is shown Figure 5C. Data are representative of three biologically independent experiments. Errors bars represent SD. $^{**}p<0.01$ (Student's t-test).

(C) Heat map of autophagy-related genes differentially expressed between shCTR and shPHF8 NSCs, identified by RNA-seq. Genes shown have a p-value < 0.05 and a $\log_2$ fold change (FC) > 0.5 or < -0.5.

APPENDIX TABLES

Appendix Table S1: List of genome-wide data accessions used in this study

Data	Accession number
RNA-seq PHF8	GSE296528
ATAC-seq	GSE296528
H4K20me1 ChIP-seq (NSC)	GSE296528
H3K4me3 ChIP-seq	GSM605316
H3K9me2 ChIP-seq	GSM3315714
H4K20me1 ChIP-seq	GSM789284
PHF8 ChIP-seq (hESC)	GSM1003509

PHF8 ChIP-seq (K562)	GSM831009
NFIA ChIP-seq	GSM2574788
NFIA RNA-seq	GSE124729
ATF4 RNA-seq	GSE291048
ATF4 ChIP-seq	GSE127432
H3K9me3 ChIP-seq	GSE311637

Appendix Table S2: Read count illustrating ChIP-seq mapping statistics

Sample	Read count				
	Raw	Mapped Reads	NoMap	UniMap	MultiMap
shCTR_input	38307439	36930220	1377219	29077062	7853158
shPHF8_input	38507414	37082909	1424505	28604086	8478823
shCTR_ChIP H3K9me3_R1	38326941	36968871	1358070	24487187	12481684
shCTR_ChIP H3K9me3_R2	37214264	35970838	1243426	24372394	11598444
shCTR_ChIP H4K20me1_R1	38262631	37648445	614186	32561439	5087006
shCTR_ChIP H4K20me1_R2	34861102	34103671	757431	29241204	4862467
shPHF8_ChIP H3K9me3_R1	38018502	36832908	1185594	24096483	12736425
shPHF8_ChIP H3K9me3_R2	32969097	31654353	1314744	19934294	11720059
shPHF8_ChIP H4K20me1_R1	38436855	37797844	639011	32780889	5016955
shPHF8_ChIP H4K20me1_R2	19471153	18999554	471599	16114507	2885047
