# Supplementary figures and images for: Epigenetic regulation of serine biosynthesis by PHF8 during neurogenesis
Source: EMBO Rep. 2026 Feb 19;27(6):1540–60. doi: 10.1038/s44319-026-00713-8 (PMC13022353; doi:10.1038/s44319-026-00713-8)

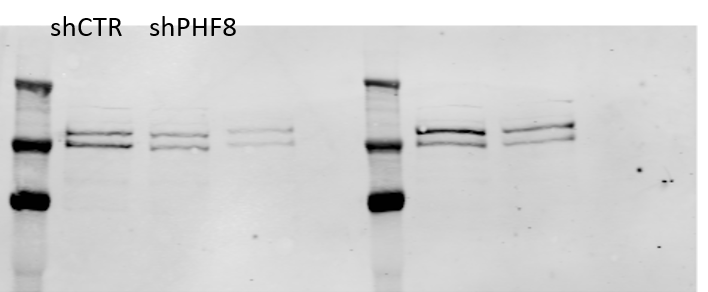

Supplement: Supplementary file 3 — Source data Fig. 1 [file 44319_2026_713_MOESM3_ESM.zip › Fig 1/1B/1B_western blot PHF8.tif]

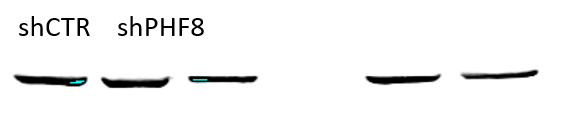

Supplement: Supplementary file 3 — Source data Fig. 1 [file 44319_2026_713_MOESM3_ESM.zip › Fig 1/1B/1B_western blot Tub.tif]

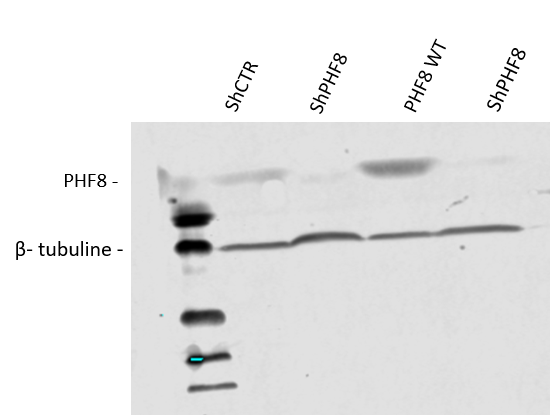

Supplement: Supplementary file 4 — Source data Fig. 2 [file 44319_2026_713_MOESM4_ESM.zip › Fig 2/2F/WB.tif]

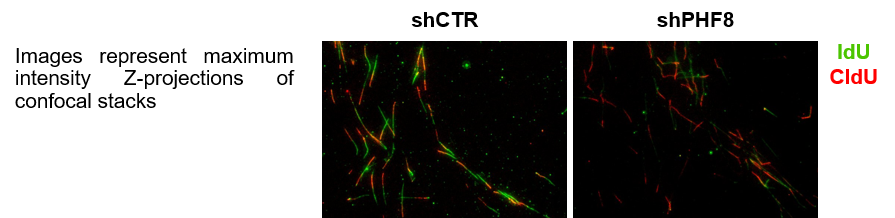

Supplement: Supplementary file 5 — Source data Fig. 4 [file 44319_2026_713_MOESM5_ESM.zip › Fig 4/4 E/Images DNA fiber.tif]

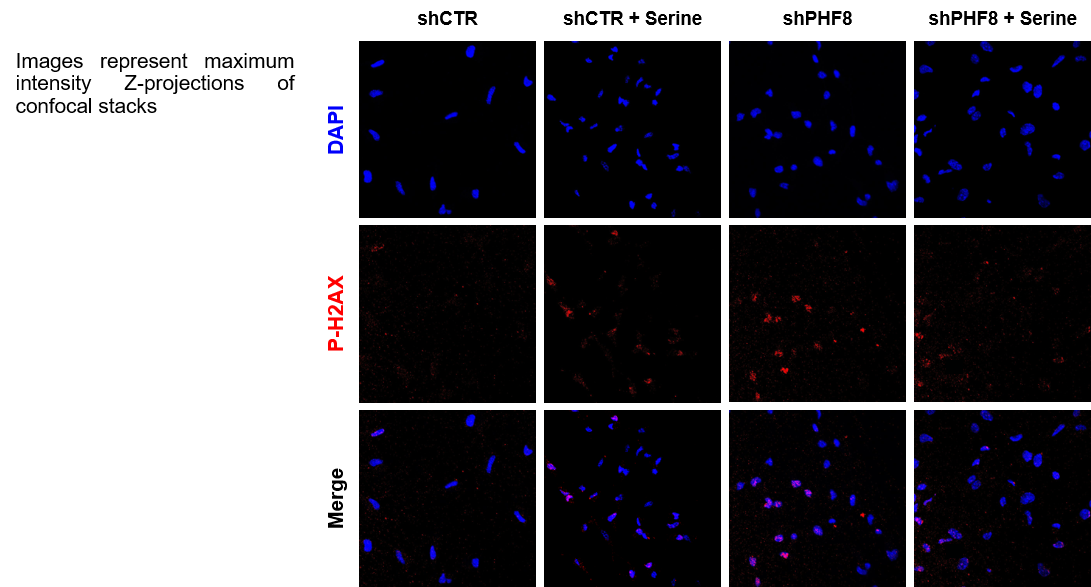

Supplement: Supplementary file 5 — Source data Fig. 4 [file 44319_2026_713_MOESM5_ESM.zip › Fig 4/4 F/IF P-H2AX.tif]

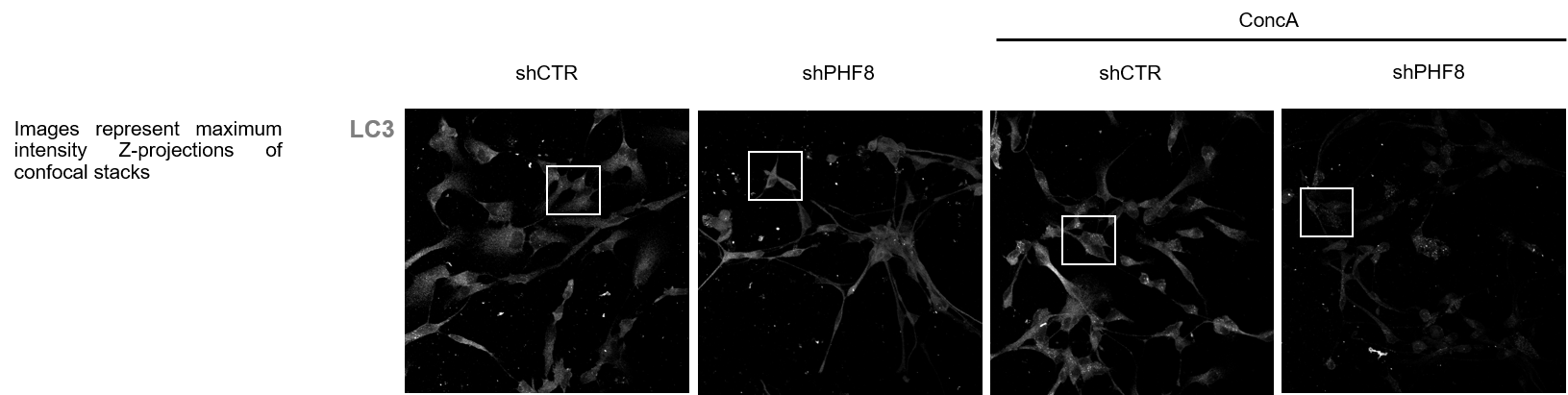

Supplement: Supplementary file 6 — Source data Fig. 5 [file 44319_2026_713_MOESM6_ESM.zip › Fig 5/5B/IF Fig 5 B.tif]

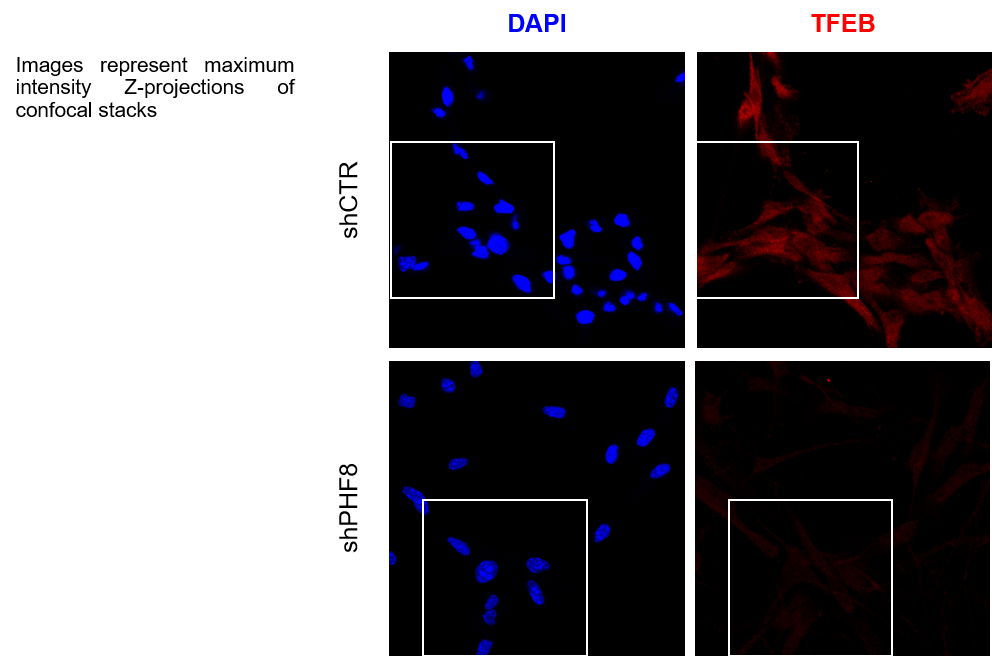

Supplement: Supplementary file 6 — Source data Fig. 5 [file 44319_2026_713_MOESM6_ESM.zip › Fig 5/5D/IF Fig 5 D.tif]

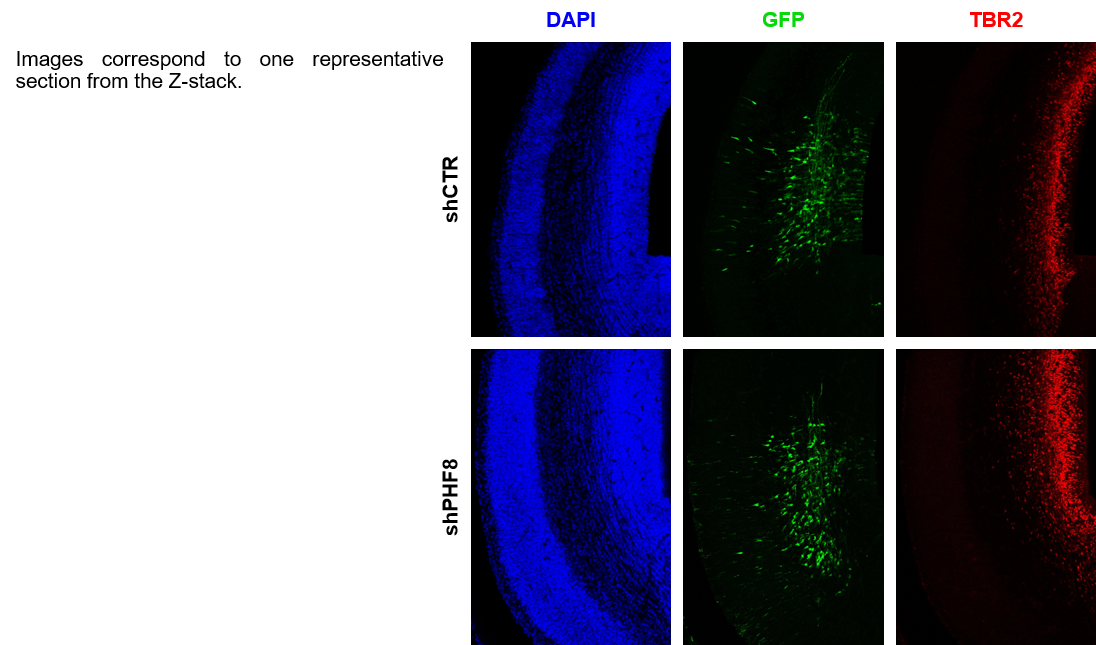

Supplement: Supplementary file 7 — Source data Fig. 6 [file 44319_2026_713_MOESM7_ESM.zip › Fig 6/6A/IF Fig 6A.tif]

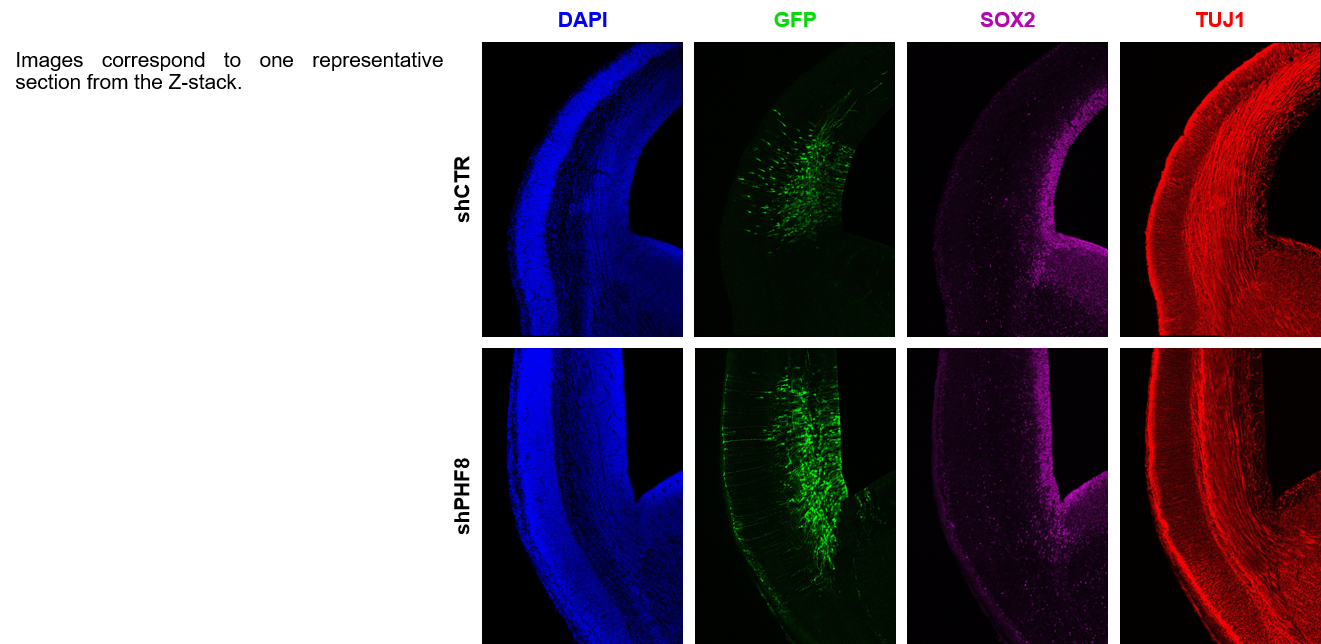

Supplement: Supplementary file 7 — Source data Fig. 6 [file 44319_2026_713_MOESM7_ESM.zip › Fig 6/6B/IF Fig 6B.tif]

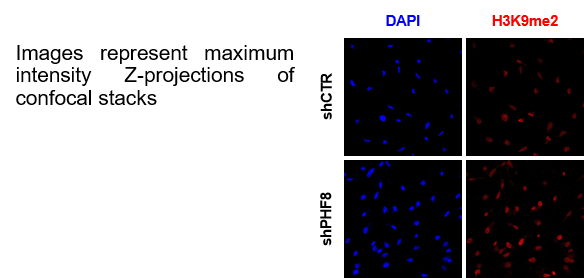

Supplement: Supplementary file 8 — EV Figures Source Data [file 44319_2026_713_MOESM8_ESM.zip › EV Figures/Fig EV1/IF H3K9me2.tif]

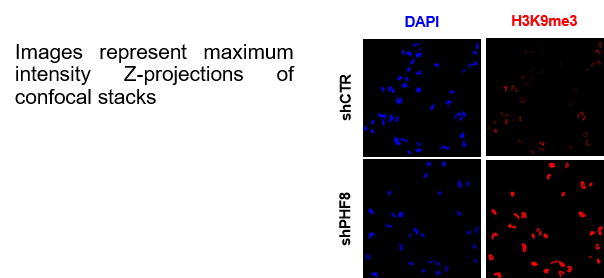

Supplement: Supplementary file 8 — EV Figures Source Data [file 44319_2026_713_MOESM8_ESM.zip › EV Figures/Fig EV1/IF H3K9me3.tif]

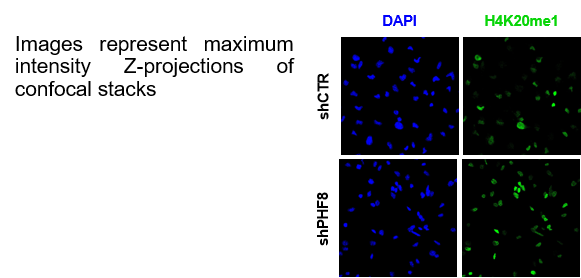

Supplement: Supplementary file 8 — EV Figures Source Data [file 44319_2026_713_MOESM8_ESM.zip › EV Figures/Fig EV1/IF H4K20me1.tif]

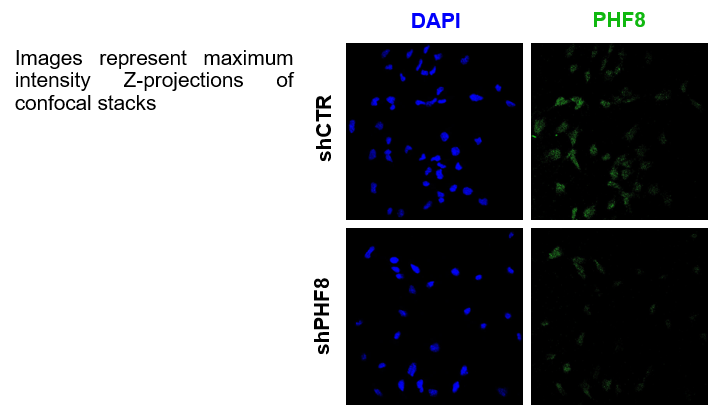

Supplement: Supplementary file 8 — EV Figures Source Data [file 44319_2026_713_MOESM8_ESM.zip › EV Figures/Fig EV1/IF PHF8.tif]

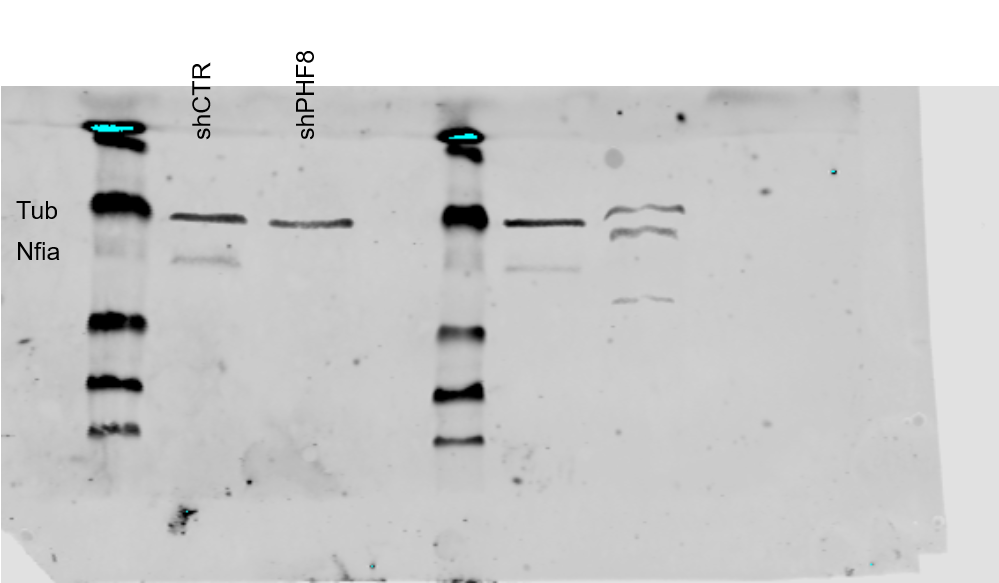

Supplement: Supplementary file 8 — EV Figures Source Data [file 44319_2026_713_MOESM8_ESM.zip › EV Figures/Fig EV2/EV2 D/EV2-D_NFIA and tub western blot.png]

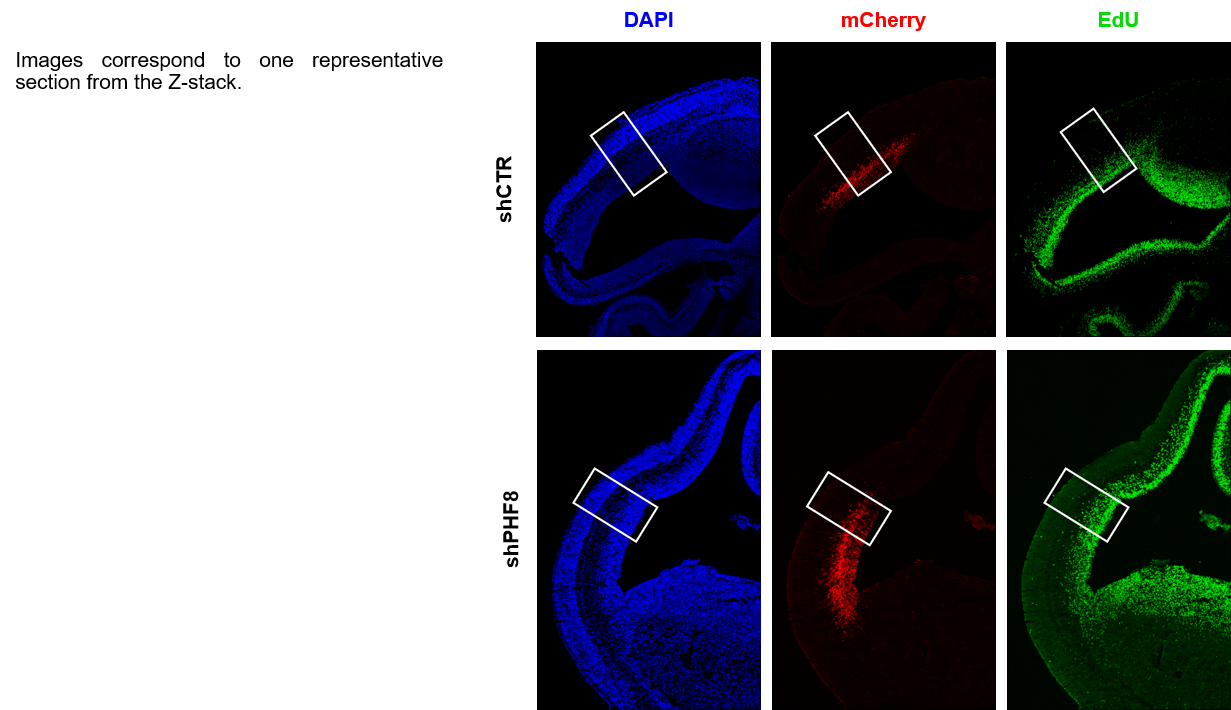

Supplement: Supplementary file 8 — EV Figures Source Data [file 44319_2026_713_MOESM8_ESM.zip › EV Figures/Fig EV3/EV3 A-B/IF EV3 A-B.tif]

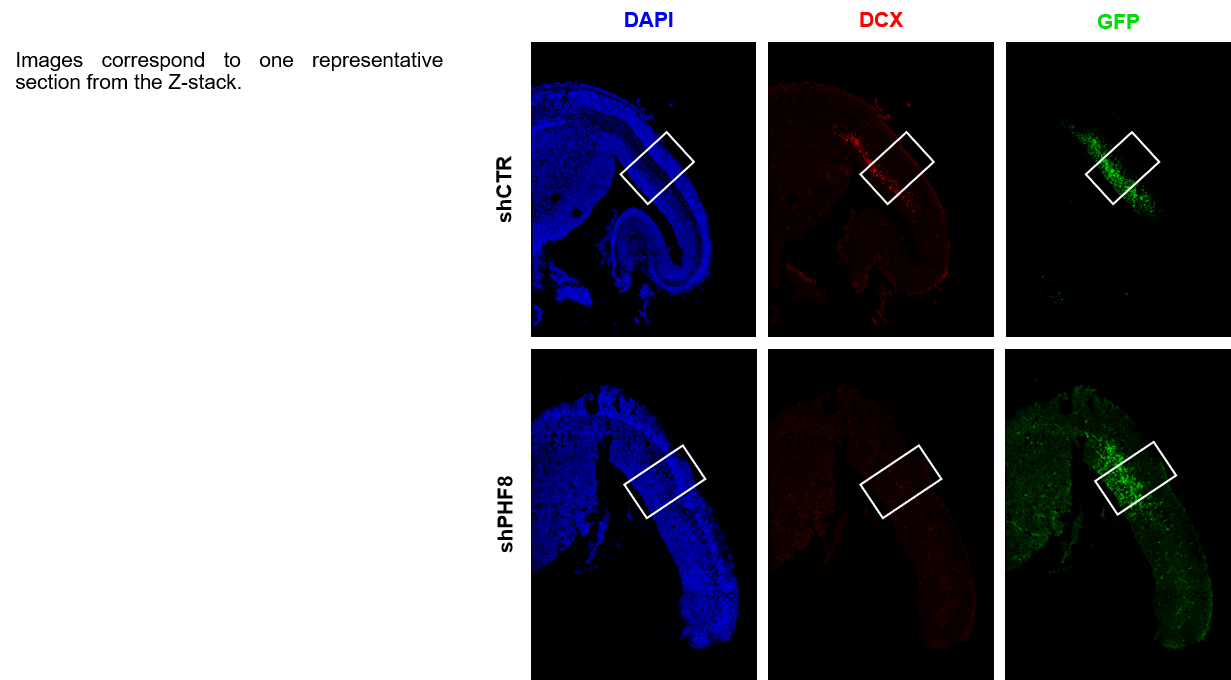

Supplement: Supplementary file 8 — EV Figures Source Data [file 44319_2026_713_MOESM8_ESM.zip › EV Figures/Fig EV3/EV3 D/IF EV3 D.tif]
