# Supplementary material for: Epigenetic regulation of serine biosynthesis by PHF8 during neurogenesis
Source: EMBO Rep. 2026 Feb 19;27(6):1540–60. doi: 10.1038/s44319-026-00713-8 (PMC13022353; doi:10.1038/s44319-026-00713-8)

CMM_002_WB autophagic Flux

Figure 5A
PONCEAU

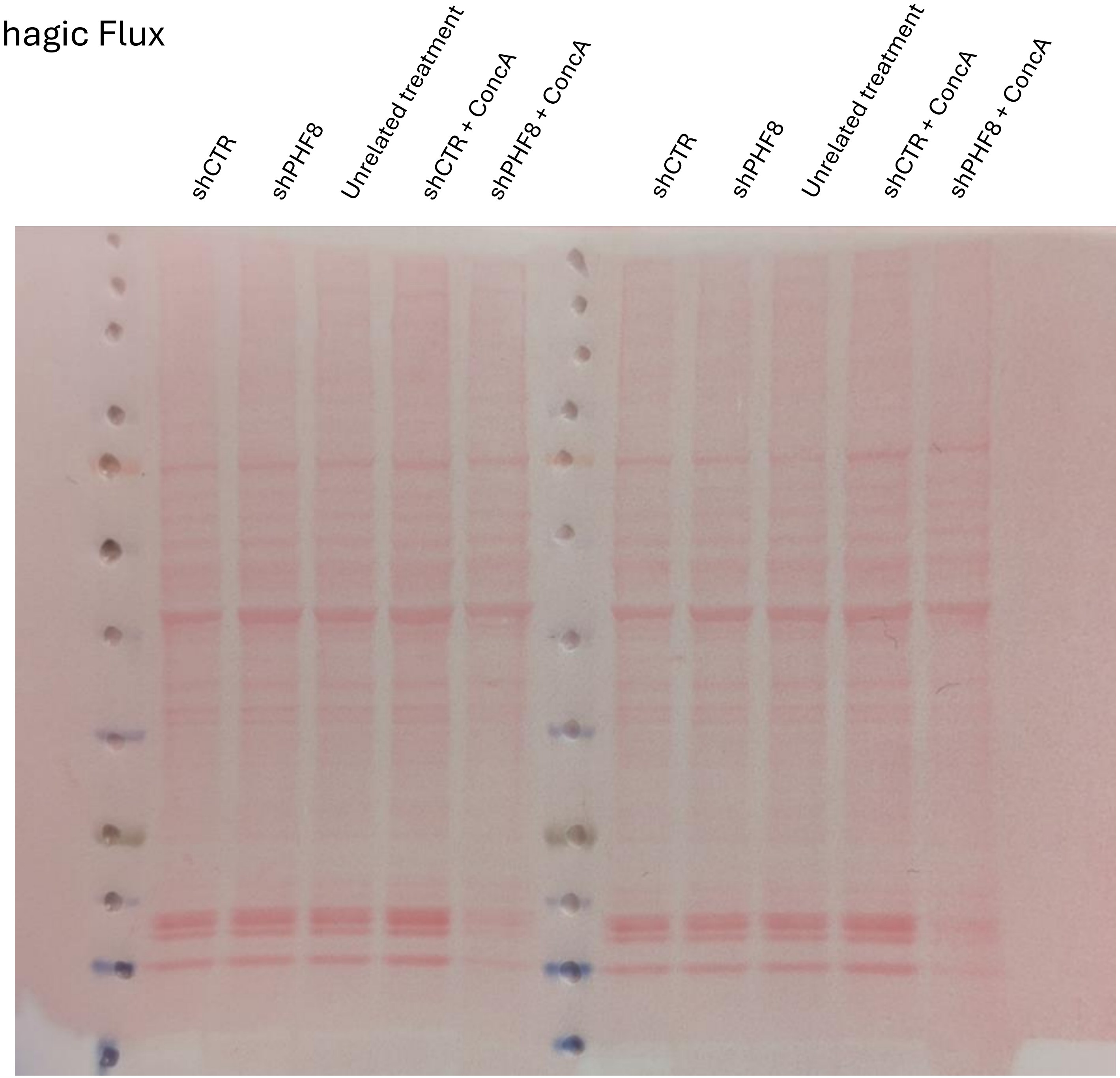

Figure 5A

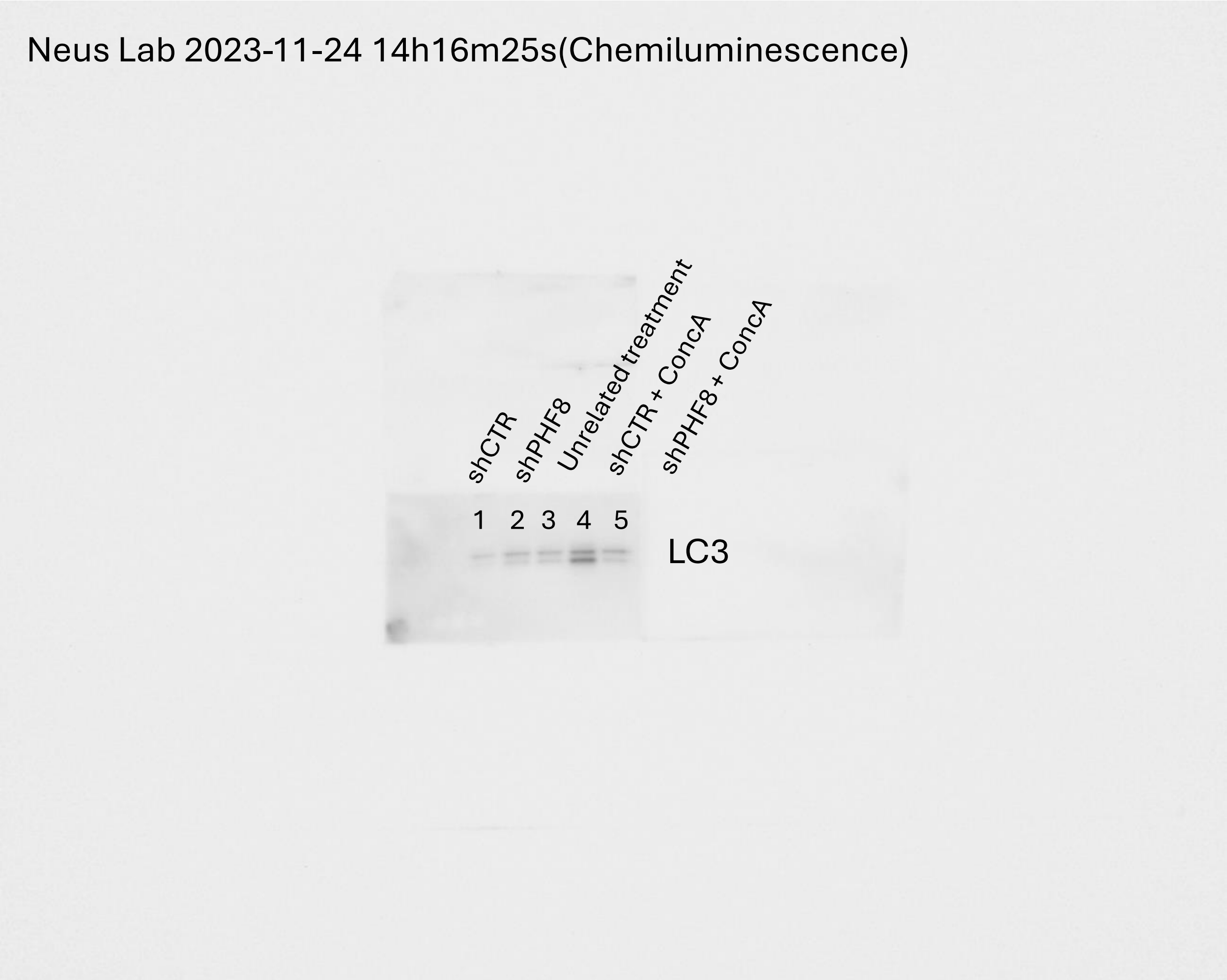
Neus Lab 2023-11-24 14h16m25s(Chemiluminescence)
shCTR
shPHF8
Unrelated treatment
shCTR + ConcA
shPHF8 + ConcA
1 2 3 4 5
LC3

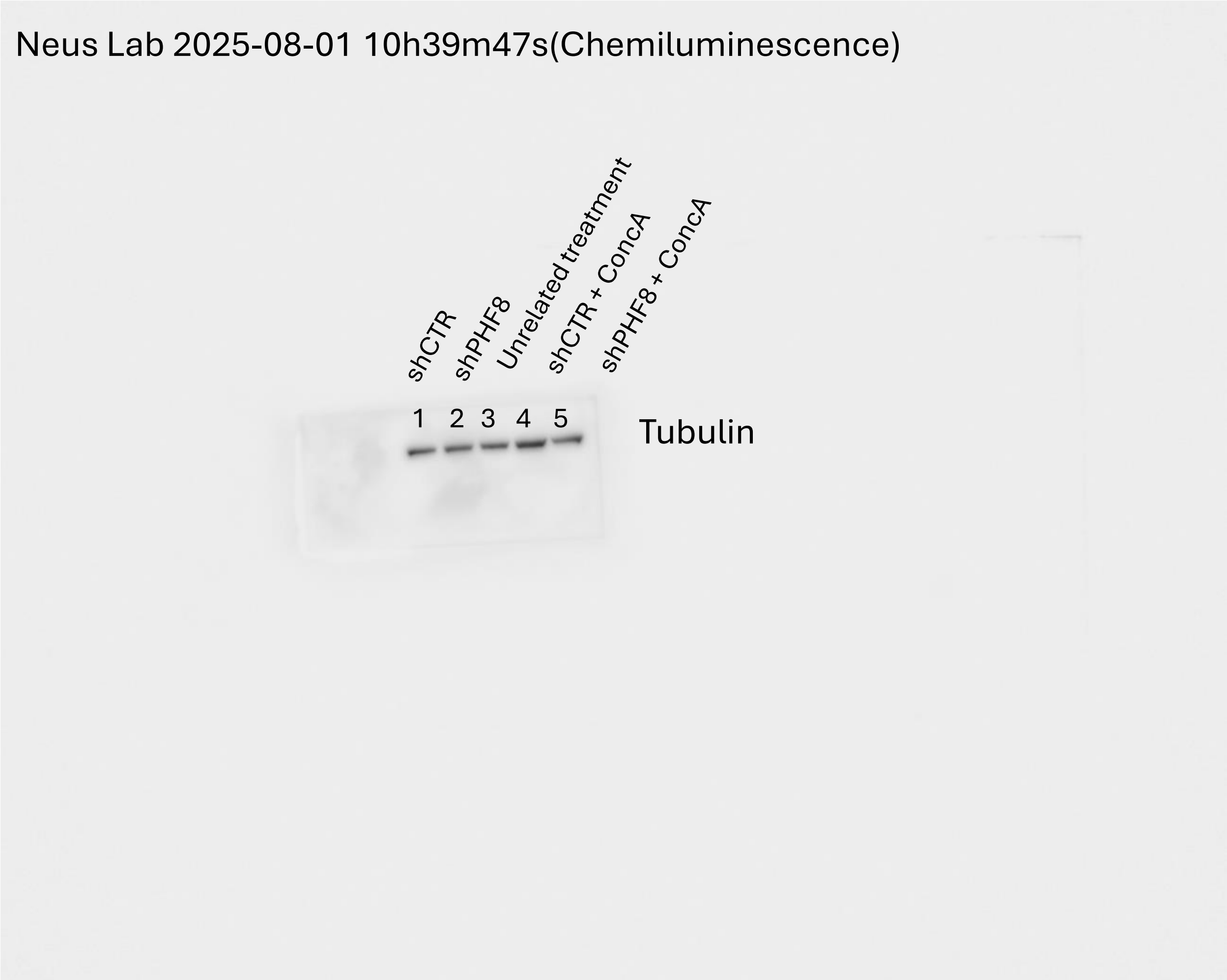
Neus Lab 2025-08-01 10h39m47s(Chemiluminescence)
shCTR
shPHF8
Unrelated treatment
shCTR + ConcA
shPHF8 + ConcA
1 2 3 4 5
Tubulin

CMM_002_mTOR activity

Figure 5C
PONCEAU

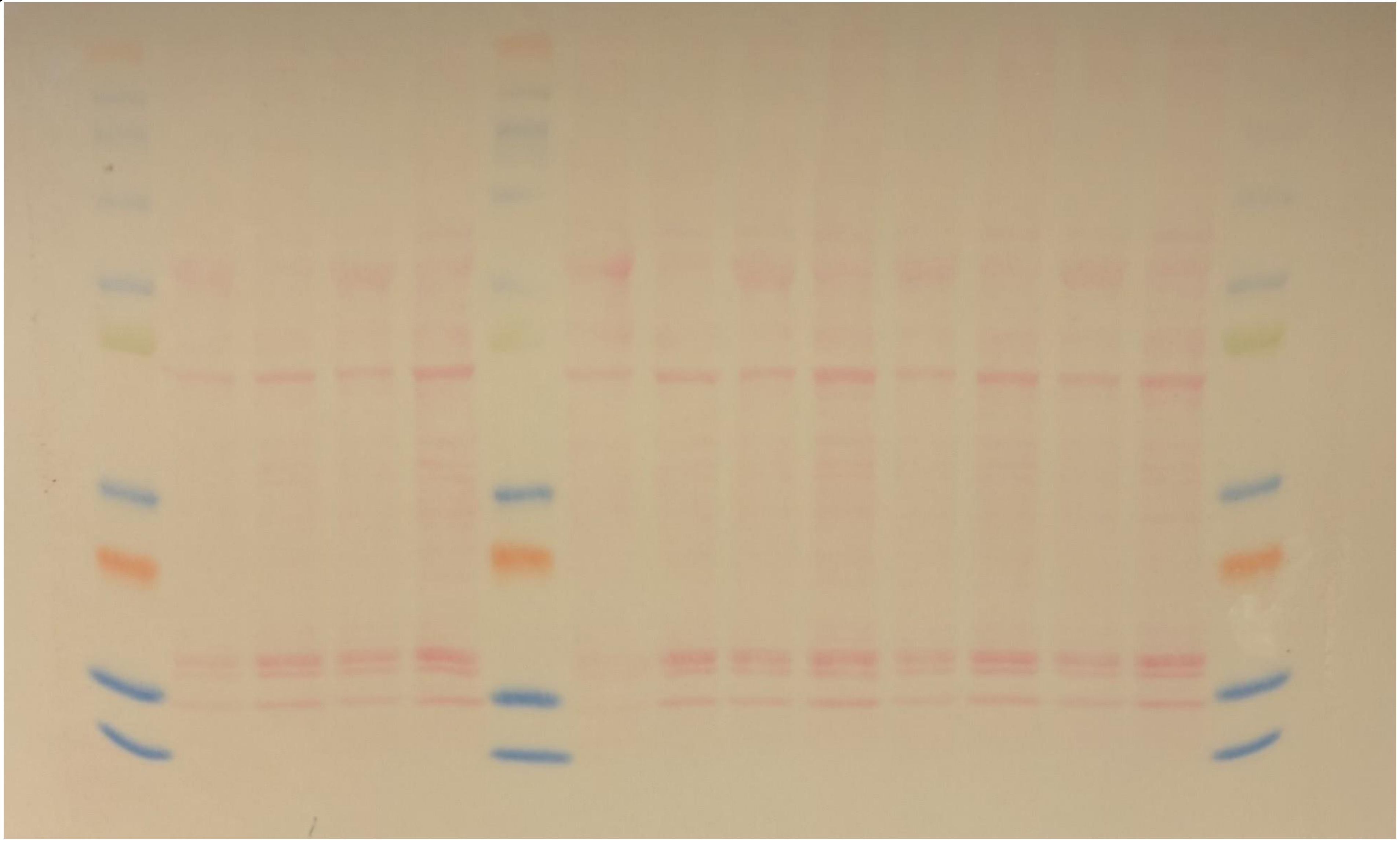

CMM_002_mTOR activity

Figure 5C

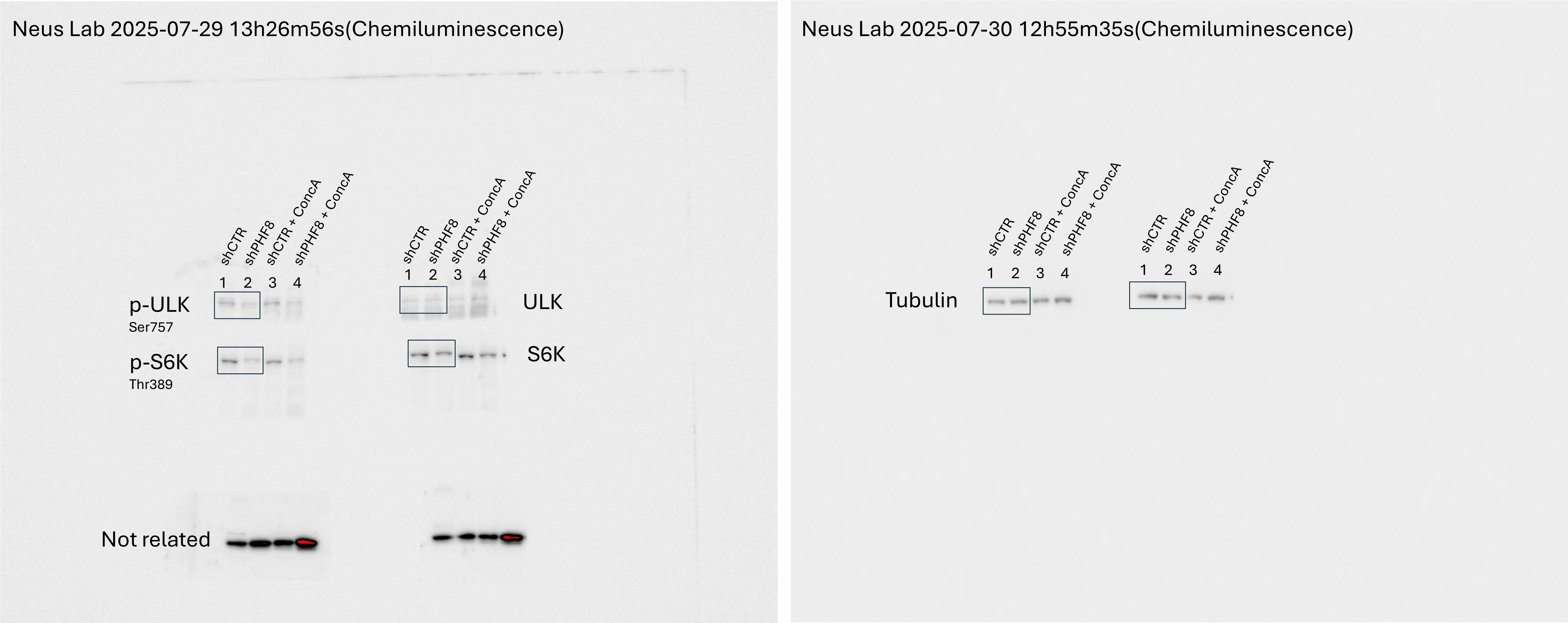

Supplement: Supplementary file 6 — Source data Fig. 5 [file 44319_2026_713_MOESM6_ESM.zip › Fig 5/5A/WB fig 5 A-C.pdf]
