# Supplementary material for: Epigenetic regulation of serine biosynthesis by PHF8 during neurogenesis
Source: EMBO Rep. 2026 Feb 19;27(6):1540–60. doi: 10.1038/s44319-026-00713-8 (PMC13022353; doi:10.1038/s44319-026-00713-8)
Supplement: Supplementary file 9 — Expanded View Figures [file 44319_2026_713_MOESM9_ESM.pdf]

Expanded View Figures

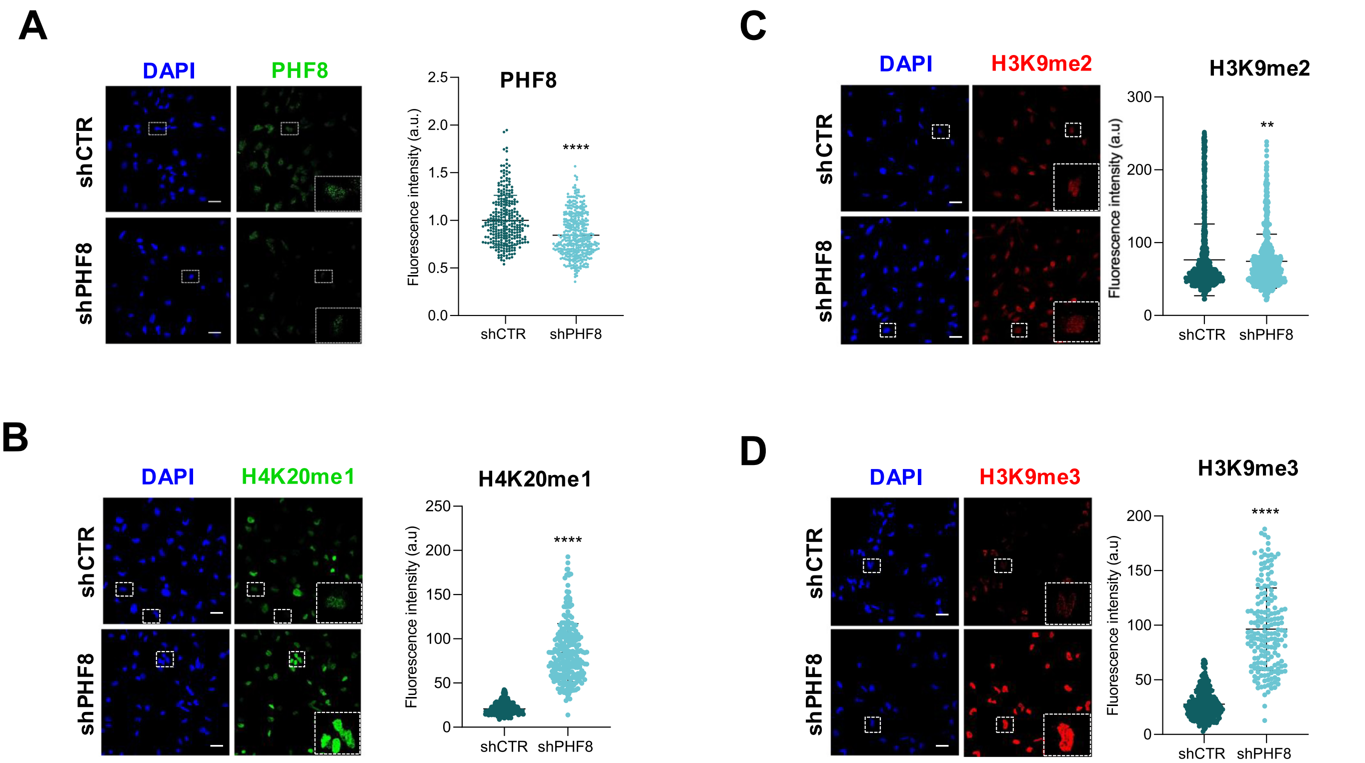

Figure EV1. PHF8 maintains transcriptionally competent chromatin.

(**A–D**) shCTR and shPHF8 NSCs were immunostained using PHF8 (**A**), H4K20me1 (**B**), H3K9me2 (**C**), and H3K9me3 (**D**) antibodies and DAPI. Violin plots show quantification of the fluorescence intensity normalized to cell area ($n \geq 180$). The data shown are representative of three biologically independent experiments. Scale bar indicates 20 μm. Error bars represent mean ± SEM. ***p* = 0.0036; *****p* < 0.0001, two-tailed *t*-test. Source data are available online for this figure.

© The Author(s)

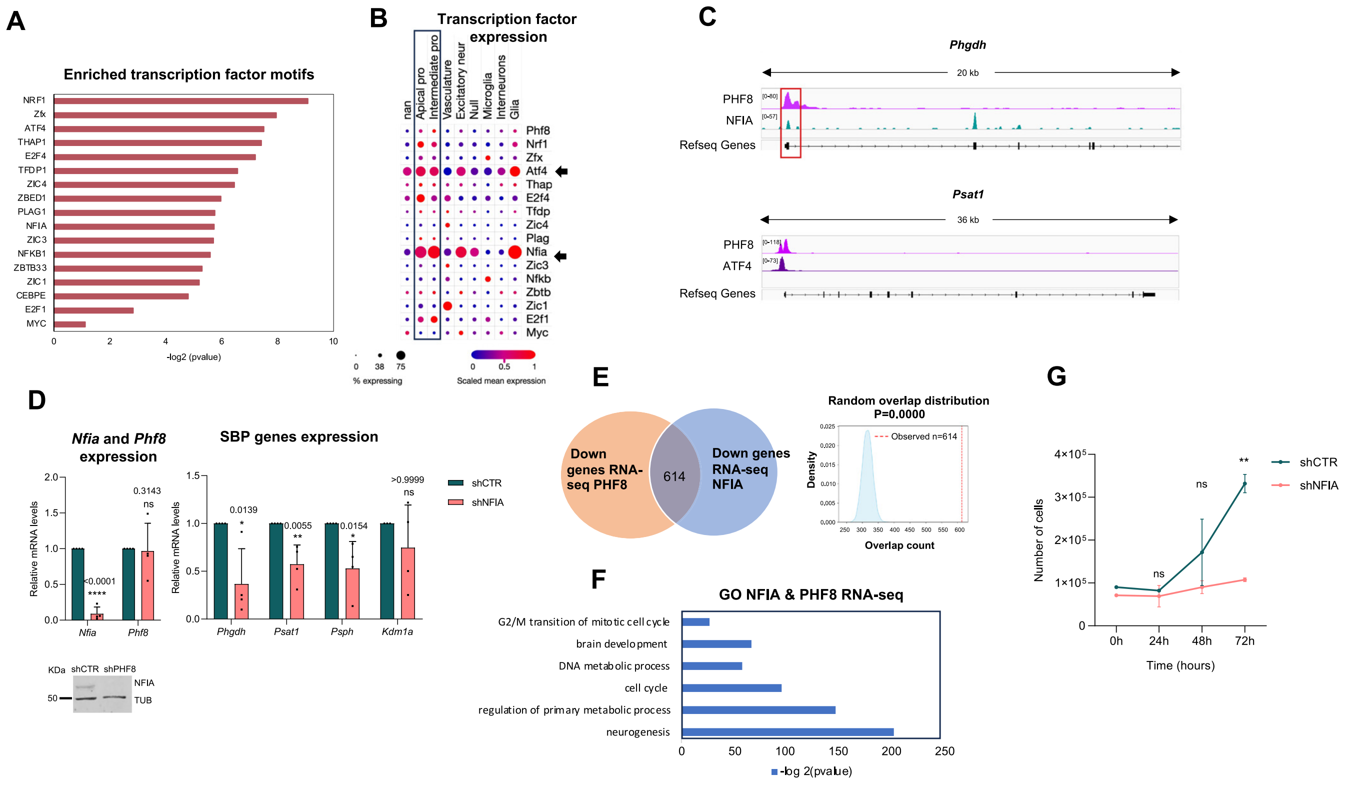

Figure EV2. PHF8 cooperates with transcription factors to regulate SBP gene transcription.

(**A**) Motif enrichment analysis of promoters from metabolism-related, PHF8-regulated genes was performed using the PSCAN tool, the top enriched transcription factor motifs. Z-test implemented in PSCAN. (**B**) Graph showing the expression of *Phf8* and the transcription factors identified in (**A**) in the developing mouse cerebral cortex. Data are derived from publicly available single-cell RNA-seq datasets (link to dataset). (**C**) IGV snapshots showing PHF8 binding peaks, NFIA and ATF4 peaks at *Phgdh* (NFIA) and *Psat1* (ATF4) gene promoters. Tracks show the input-subtracted signal. (**D**) NSCs were infected with lentiviruses expressing shCTR or NFIA-targeting shRNA (shNFIA). Total RNA and protein extracts were collected to assess NFIA protein levels via immunoblotting and mRNA levels of *Nfia*, *Phf8*, *Phgdh*, *Psat1*, and *Psph* by qPCR. Expression levels from four biologically independent experiments were normalized to *Gapdh*, and data were presented relative to shCTR samples. *Kdm1a* was included as a negative control. Error bars represent mean ± SEM. Two-tailed *t*-test was applied. (**E**) Venn diagram showing the overlap between genes downregulated in our PHF8 KD RNA-seq (Fig. 2) (orange) and genes downregulated upon NFIA knockdown identified in the published NFIA KD RNA-seq dataset (Appendix Table S1) (blue). p-value < 0.05. Graph showing the median differences from the permutation test between PHF8 downregulated genes in the RNA-seq dataset and 2048 randomly selected genes (corresponding to the number of genes downregulated in NFIA KD). A total of $n = 10{,}000$ permutations were performed. The observed overlap value (614) (red vertical bar) was not reached in any of the random samples, resulting in an empirical p value of zero (right panel). (**F**) GO analysis highlighting enriched Biological Processes among genes co-regulated by PHF8 and NFIA identified in (**E**). p value <0.05. Enrichment was assessed by Fisher's exact test, with P values corrected using the g:SCS method in g:Profiler. (**G**) Growth curve showing proliferation rates of NSCs infected with shCTR or shNFIA lentiviruses over 72 h. Data represent the mean of three biologically independent experiments performed in triplicate. Error bars represent SD. $^{**}p = 0.0040$; ns not significant, two-tailed t-test. Source data are available online for this figure.

 © The Author(s)

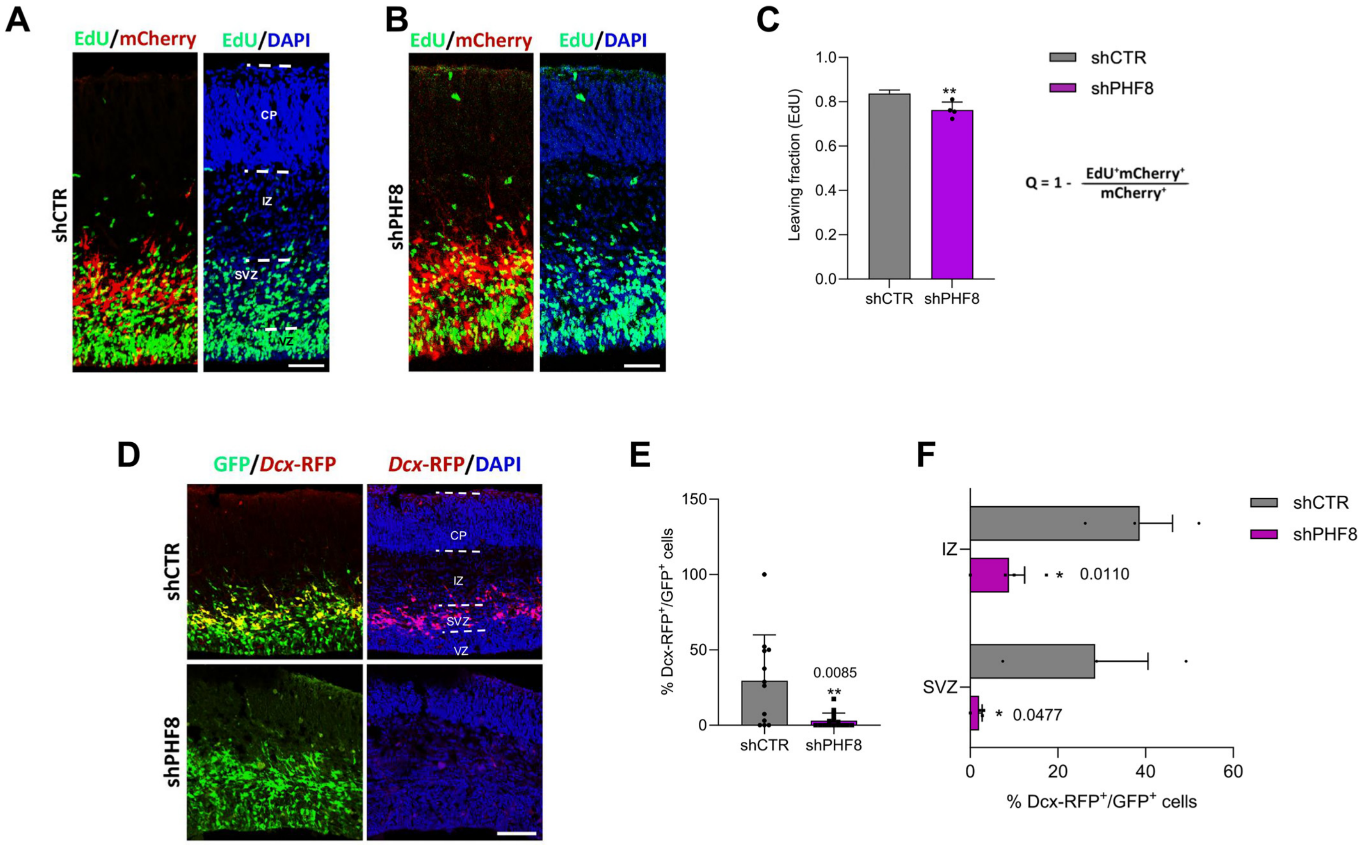

Figure EV3. PHF8 depletion reduces mouse neurogenesis in vivo and impairs the differentiation of the neuronal outputs.

(**A**, **B**) Representative images of E16.5 brain sections from embryos electroporated in utero at E14.5 with either shCTR or shPHF8 constructs, together with an mCherry reporter. EdU was administered to pregnant dams 34 h post-IUE, and embryos were collected 14 h later for brain dissection. Data represent the mean from four to twelve embryos (derived from at least three shCTR and four shPHF8 biologically independent experiments). Scale bar: 50 µm. CP cortical plate, IZ intermediate zone, SVZ subventricular zone, VZ ventricular zone. (**C**) Quantification of the global leaving fraction (Q), estimating cell-cycle exit over the 48 h period. Q was significantly reduced in shPHF8-electroporated embryos compared with shCTR. EdU^+ cells and double $EdU^+mCherry^+$ cells were counted in the VZ–SVZ and IZ of shCTR and shPHF8-electroporated embryos. Data represent mean ± SEM from $n \geq 3$ shCTR and $n = 4$ shPHF8 biologically independent experiments. $^{**}p = 0.0096$, two-tailed *t*-test. (**D**) Analysis of the early neuronal marker DCX. E14.5 embryos were electroporated with shCTR or shPHF8 together with a GFP-expressing plasmid and a *Dcx* promoter-driven DsRed reporter construct. Data represent the mean from four to twelve embryos (derived from at least three shCTR and four shPHF8 biologically independent experiments). Scale bar: 100 µm. (**E**, **F**) Brains were dissected 48 h after electroporation. Quantification of RFP^+/GFP^+ cells, shown as total counts (**E**) and by cortical region (SVZ and IZ) (**F**), revealed a significant reduction in *Dcx*-RFP reporter expression in the shPHF8 condition compared with shCTR. Data represent mean ± SEM from $n \geq 3$ shCTR and $n = 4$ shPHF8 biologically independent experiments. Two-tailed *t*-test was applied. Source data are available online for this figure.
